# ABO blood group relationships to kidney transplant recipient and graft outcomes

**DOI:** 10.1371/journal.pone.0236396

**Published:** 2020-07-23

**Authors:** Monica S. Y. Ng, Shahid Ullah, Gregory Wilson, Stephen McDonald, Matthew Sypek, Andrew J. Mallett

**Affiliations:** 1 Kidney Health Service, Royal Brisbane and Women’s Hospital, Brisbane, Queensland, Australia; 2 Faculty of Medicine, The University of Queensland, Brisbane, Queensland, Australia; 3 Australia and New Zealand Dialysis and Transplant Registry, South Australian Health and Medical Research Institute, Adelaide, South Australia, Australia; 4 College of Medicine and Public Health, Flinders University, Adelaide, South Australia, Australia; 5 Adelaide Medical School, The University of Adelaide, Adelaide, South Australia, Australia; 6 The Department of Nephrology, The Princess Alexandra Hospital, Brisbane, Queensland, Australia; 7 Faculty of Medicine, Dentistry and Health Sciences, University of Melbourne, Melbourne, Victoria, Australia; 8 Department of Nephrology, Royal Melbourne Hospital, Melbourne, Victoria, Australia; 9 Institute for Molecular Bioscience, The University of Queensland, Brisbane, Queensland, Australia; Imperial College Healthcare NHS Trust, UNITED KINGDOM

## Abstract

**Introduction:**

Certain ABO blood types have been linked to cardiovascular disease, infection and cancers. The effect of recipient ABO blood group on patient and graft survival has not been studied in ABO-matched kidney transplantation. This study aims to determine the association between kidney transplant recipient ABO blood groups with patient and graft survival in Australian and New Zealand.

**Methods:**

All Australian and New Zealand transplant recipients who received ABO-compatible primary kidney transplants between 1995–2016 were analysed using a de-identified dataset from the Australia and New Zealand Dialysis and Transplant (ANZDATA) Registry. Primary analysis was undertaken of recipient ABO blood group O versus non-O blood groups. The primary outcome was patient survival post kidney transplantation and the secondary outcome was death censored graft survival. Recipient age at first transplant, gender, ethnicity, body mass index, smoking status, vascular disease, presence of diabetes mellitus, chronic lung disease, primary kidney disease, donor source, donor age and gender, and era of transplants were included in the multivariate model as confounders.

**Results and conclusions:**

On analysis of 15,523 kidney transplant recipients, blood group O was not associated with patient survival (hazard ratio (HR) 0.96, 95% confidence interval (CI) 0.89–1.04) nor death censored graft survival (HR 0.97, 95% CI 0.89–1.05) compared to non-blood group O recipients. Competing risks analyses showed an increased risk of cancer-related mortality in blood group O recipients on univariate analyses (HR 1.18, 95% CI 1.01–1.37) however, this became insignificant on multivariate analyses. On secondary analyses, recipient blood group AB (4.11% participants) was associated with inferior death censored graft survival compared to those with blood group O (HR 1.24, 95% CI 1.02–1.50). Although recipient ABO blood groups were not associated with patient nor graft survival, differences in cause-specific mortality between individual blood groups cannot be excluded based on current analyses.

## Introduction

The ABO blood group system consists of complex carbohydrate molecules expressed on the extracellular surface of red blood cell membranes [1]. The primary structure of these antigens is a glycoprotein or glycolipid backbone called the H structure. The A and B alleles encode for different glycosyltransferases which attach specific monosaccharides (A = *N*-acetylgalactosamine, B = D-galactose) to the H structure to form the A and B antigens [2]. The O allele do not encode a functional enzyme so the O antigen consists of the unaltered H structure. Notably, ABO antigens are also expressed on epithelium, sensory neurons, platelets and vascular endothelium [3, 4].

It follows that ABO blood group had implications for cardiovascular disease, cancer and infections [5]. Non-O blood type individuals have increased risks of venous thrombosis, peripheral vascular disease, coronary heart disease, myocardial infarction and ischaemic stroke [6, 7]. This has been attributed to increased von Willebrand’s factor half-life associated with A and B antigens; as well as higher levels of vWF and factor VIII in non-O blood type individuals [5, 8, 9]. Blood group O was associated with increased prevalence of skin cancers and non-Hodgkin’s lymphoma [10–12]. Blood group O has been associated with increased risks of disease progression in patients with bladder cancer [13, 14]. Lastly, blood group O has been correlated to increased risk of cholera, mumps and tuberculosis; while blood group A are at increased risk of *Pseudomonas aeruginosa* infections [15, 16]. This has been attributed to the role of blood group antigens as receptors and/or coreceptors for pathogens and facilitate intracellular uptake [16].

To date, no study has investigated the relationship between ABO blood group and ABO-compatible kidney transplant outcomes despite persistent differences in transplantation wait-time between different ABO blood groups. This study seeks to complete an analysis of kidney transplants conducted in Australia and New Zealand between 1995–2016 to determine the effect of ABO blood groups on patient survival and identify other interacting factors may impact on post-transplant outcomes. Lastly, this study will review patient factors that could contribute to ABO blood group-related decrements in patient survival.

## Materials and methods

### Data collection

All Australian and New Zealand transplant recipients who received ABO-compatible primary kidney transplants (living and deceased) between 1995–2016 were analysed using a de-identified dataset from the Australia and New Zealand Dialysis and Transplant (ANZDATA) Registry. ABO-compatible kidney transplants included ABO-matched, donor blood group O and recipient blood group AB transplants (S1 Table). All transplanting units in Australia and New Zealand contribute to the ANZDATA Registry. Research was approved by ANZDATA executive (ID 42058) and Royal Adelaide Hospital Human Research Ethics Committee (HREC/17/RAH/408 R20170927). Written consent was not obtained as all data received from ANZDATA was fully anonymized prior to analysis.

### Statistical analysis

The main exposure variable was the cohort identified by recipient ABO blood group: blood group O versus non-blood group O. Secondary analyses compared each ABO blood group to blood group O. Covariates included: recipient’s age at first transplant, gender, ethnicity, body mass index (BMI), smoking status, vascular disease, presence of diabetes mellitus, chronic lung disease, primary kidney disease; donor source, age, ethnicity and gender, and era of transplants (1995–1999, 2000–2004, 2005–2009, 2010–2015). Comorbidities, BMI and smoking status were recorded at time of transplant. Vascular disease was categorised by the presence of at least one of ischemic heart disease, cerebrovascular disease and/or peripheral vascular disease. Ethnicity and gender were classified by patient’s self-identification on registration forms. Primary renal disease were as identified by treating clinicians on ANZDATA data entry forms based on renal biopsy results and/or clinical features.

The primary outcome was patient survival post kidney transplantation and the secondary outcome was death censored graft survival. Patient survivals were measured from the date of transplant to the date of death, patients were censored at date of loss to follow-up or 31st of December 2016. Death censored graft survivals were calculated from the date of transplant to the date of irreversible graft failure signified by long-term dialysis (or re-transplantation) or the date of last follow-up during the period when the graft was still functioning. In the event of death with a functioning graft, the follow-up period is censored at the date of death.

All statistical analyses were performed using Stata statistical software, version 15.1. Patient’s characteristics at transplant were expressed as median and intra quartile range (IQR) for continuous skewed data and proportions were presented as percentages of the respective denominator.

Cox proportional hazard models were applied to examine the survival outcomes between groups. Multivariate modelling was then undertaken by adding previously listed covariates. Transplant waiting times were included as a continuous variable. The estimates were calculated using the likelihood ratio method and were expressed as hazard ratios (HRs). Proportional hazard assumption was tested by log-log plot of survival and Schoenfeld Residuals. Survival curves between blood groups were evaluated by standard Kaplan–Meier survival curves and groups were compared by log-rank test. The departure from linearity for continuous predictors was tested by linear spline for the adaptive model and found an evidence that the assumption of linearity had been violated (Wald test p-values >0.05). Linear splines with knots at specified points were applied for non-linear relationships.

Competing risk analysis was performed on causes of survival outcomes between groups. In the competing risk set up, under each cause for the occurrence of an event of interest, a hazard function in the presence of confounders is considered. The causes other than the cause of interest are considered as competing events. Survival times are defined as the time until occurrence of one competing event preventing other event to occur. Fine and Gray subdistribution hazards models were to assess the sub-distribution hazard ratios (SHR) of cause specific mortality [17].

## Results

### Demographic characteristics

15,523 patients received primary kidney transplants between 1995 to 2016. Blood group O comprised of 44.1% of recipients (n = 6,839), blood group A comprised of 39.7% (n = 6,166), blood group B comprised of 11.4% (n = 1,769) and blood group AB comprised of 4.1% (n = 639) (Table 1). Recipients with blood group B and AB were more likely to be Asian than white compared to blood groups O and A recipients. Median dialysis duration was the shortest for recipients with blood group A (1.6 years) and longest for blood group O (2.3 years) however, blood group AB recipients were most likely to receive a graft within 5 years (95.2%). Median total ischaemia time was greatest for AB group grafts (11.0 hours). Blood group AB recipients were more likely to receive deceased donor kidneys (74.6%). Recipient age group, gender, primary renal disease, smoking status, vascular disease, diabetes, respiratory disease and transplant era were similar between recipient blood groups. Cardiovascular death (29%) was the most common cause of death in the study period followed by cancer (22%) and infection (18%).

**Table 1:** Patient characteristics at transplant between ABO groups (n = 15,523).

Characteristics	All; n (%)	ABO group			
		O; n(%)	A; n (%)	B; n (%)	AB; n(%)
N	15,523	6839 (44.1)	6,166 (39.7)	1769 (11.4)	639 (4.1)
Age at transplant, y, median (IQR)	48.0 (35.0–58.0)	48.0 (35.0–58.0)	48.0 (36.0–58.0)	48.0 (36.0–57.0)	49.0 (37.0–58.0)
Age group, y					
<15	623 (4.0)	273 (4.0)	243 (3.9)	67 (3.8)	25 (3.9)
15–44	5,954 (38.4)	2686 (39.3)	2,356 (38.2)	652 (36.9)	220 (34.4)
45–54	3,818 (24.6)	1632 (23.9)	1,522 (24.7)	469 (26.5)	173 (27.1)
55–64	3,722 (24.0)	1649 (24.1)	1,471 (23.9)	414 (23.4)	160 (25.0)
65+	1,406 (9.1)	599 (8.8)	574 (9.3)	167 (9.4)	61 (9.5)
Gender				
Male	9,658 (62.2)	4281 (62.6)	3,808 (61.8)	1097 (62.0)	402 (62.9)
Female	5,865 (37.8)	2558 (37.4)	2,358 (38.2)	672 (38.0)	237 (37.1)
BMI, kg/m2				
<18.5	492 (3.2)	218 (3.2)	170 (2.8)	78 (4.4)	22 (3.4)
18.5–24.9	6,085 (39.2)	2714 (39.7)	2,349 (38.1)	733 (41.4)	252 (39.4)
25–29.9	5,117 (33.0)	2259 (33.0)	2,062 (33.4)	554 (31.3)	214 (33.5)
> = 30	2,998 (19.3)	1265 (18.5)	1,276 (20.7)	319 (18.0)	124 (19.4)
Missing	831 (5.4%)	383 (5.6)	309 (5.0)	85 (4.8)	27 (4.2)
Ethnicity				
White	12268 (79.0)	5545 (81.1)	5,014 (81.3)	1184 (66.9)	444 (69.5)
ATSI	488 (3.1)	230 (3.4)	223 (3.6)	23 (1.3)	11 (1.7)
Asian	1,520 (9.8)	579 (8.5)	389 (6.3)	426 (24.1)	121 (18.9)
Maori	404 (2.6)	153 (2.2)	216 (3.5)	18 (1.0)	15 (2.3)
Pacific	439 (2.8)	171 (2.5)	176 (2.9)	62 (3.5)	22 (3.4)
Other	287 (1.8)	118 (1.7)	104 (1.7)	44 (2.5)	21 (3.3)
Missing	117 (0.9)	43 (0.6)	44 (0.7)	12 (0.7)	5 (0.8)
Primary renal disease					
Diabetic Nephropathy	2,157 (13.9)	928 (13.6)	869 (14.1)	242 (13.7)	107 (16.7)
Glomerulonephritis	6,624 (42.7)	2904 (42.5)	2,606 (42.3)	807 (45.6)	277 (43.3)
Hypertension	811 (5.2)	354 (5.2)	318 (5.2)	92 (5.2)	41 (6.4)
Polycystic Disease	2,128 (13.7)	975 (14.3)	840 (13.6)	215 (12.2)	78 (12.2)
Reflux Nephropathy	1,240 (8.0)	555 (8.1)	503 (8.2)	132 (7.5)	44 (6.9)
Other/Uncertain	2,489 (16.0)	1096 (16.0)	1,004 (16.3)	272 (15.4)	91 (14.2)
Missing	74 (0.5)	27 (0.4)	26 (0.4)	9 (0.5)	1 (0.2)
Dialysis duration, y, median (IQR)	1.9 (0.7–3.8)	2.3 (0.8–4.4)	1.6 (0.6–3.2)	2.1 (0.8–4.2)	1.2 (0.5–2.2)
Dialysis duration, y				
Pre-emptive	1,877 (12.1)	778 (11.4)	803 (13.0)	179 (10.1)	77 (12.1)
≤1	6,197 (39.9)	2,381 (34.8)	2,725 (44.2)	663 (37.5)	381 (59.6)
2–3	3,833 (24.7)	1,745 (25.5)	1,487 (24.1)	457 (25.8)	130 (20.3)
≥4	3,616 (23.3)	1,935 (28.3)	1,151 (18.7)	470 (26.6)	51 (8.0)
Smoking status					
Never	8,948 (57.6)	3942 (57.6)	3,541 (57.4)	1047 (59.2)	348 (54.5)
Former	4,833 (31.1)	2107 (30.8)	1,948 (31.6)	523 (29.6)	229 (35.8)
Current	1,590 (10.2)	715 (10.5)	629 (10.2)	181 (10.2)	58 (9.1)
Missing	152 (1.0)	75 (1.1)	48 (0.9)	18 (1.0)	4 (0.6)
Vascular disease	3,224 (20.8)	1406 (20.6)	1,290 (20.9)	374 (21.1)	144 (22.5)
Missing	64 (0.4)	27 (0.4)	22 (0.4)	8 (0.5)	3 (0.5)
Diabetes	2,839 (18.3)	1227 (17.9)	1,125 (18.2)	337 (19.1)	136 (21.3)
Missing	63 (0.4)	25 (0.4)	22 (0.4)	8 (0.5)	3 (0.5)
Respiratory disease	974 (6.3)	438 (6.4)	370 (6.0)	121 (6.8)	40 (6.3)
Missing	64 (0.4)	28 (0.4)	21 (0.3)	8 (0.5)	3 (0.5)
Total ischemia time, h, Median (IQR)	9.0 (3.0–14.0)	9.0 (3.0–14.0)	9.0 (3.0–14.0)	9.0 (4.0–14.0)	11.0 (5.0–15.0)
Total ischemia time, h					
<12h	9,249 (59.6)	4047 (59.2)	3,746 (60.8)	1055 (59.6)	319 (49.9)
12h-18h	4,511 (29.1)	1999 (29.2)	1,752 (28.4)	520 (29.4)	232 (36.3)
18h+	1,309 (8.4)	605 (8.8)	498 (8.1)	137 (7.7)	68 (10.6)
Missing	454 (2.9)	188 (2.7)	170 (2.8)	57 (3.2)	20 (3.1)
HLA mismatches					
0	970 (6.2)	428 (6.3)	360 (5.8)	82 (4.6)	23 (3.6)
1	1,291 (8.3)	662 (9.7)	484 (7.8)	114 (6.4)	31 (4.9)
2	3,163 (20.4)	1569 (22.9)	1,237 (20.1)	275 (15.5)	74 (11.6)
3	3,112 (20.0)	1432 (20.9)	1,239 (20.1)	330 (18.7)	100 (15.6)
4	2,526 (16.3)	1028 (15.0)	993 (16.1)	360 (20.4)	138 (21.6)
5	2,960 (19.1)	1098 (16.1)	1,246 (20.2)	424 (24.0)	187 (29.3)
6	1,501 (9.7)	622 (9.1)	607 (9.8)	184 (10.4)	86 (13.5)
Type of Donors					
Live	5,591 (36.0)	2485 (36.3)	2,277 (36.9)	576 (32.6)	162 (25.4)
Deceased	9,932 (64.0)	4354 (63.7)	3,889 (63.1)	1193 (67.4)	477 (74.6)
Donor age, y, median (IQR)	46.0 (31.0–56.0)	46.0 (31.0–56.0)	46.0 (32.0–57.0)	45.0 (29.0–55.0)	44.0 (29.0–56.0)
Donor age group, y					
<25	2,782 (17.9)	1264 (18.5)	1,063 (17.2)	317 (17.9)	131 (20.5)
25–34	1,711 (11.0)	718 (10.5)	687 (11.1)	220 (12.4)	76 (11.9)
35–44	2,710 (17.5)	1171 (17.1)	1,088 (17.6)	314 (17.8)	114 (17.8)
45–54	3,768 (24.3)	1669 (24.4)	1,501 (24.3)	434 (24.5)	132 (20.7)
55–64	3,133 (20.2)	1354 (19.8)	1,263 (20.5)	343 (19.4)	145 (22.7)
65+	1,392 (9.0)	648 (9.5)	558 (9.0)	139 (7.9)	41 (6.4)
Missing	27 (0.2)	15 (0.2)	6 (0.1)	2 (0.1)	-
Donor gender					
Male	7,768 (50.0)	3409 (49.8)	3,027 (49.1)	940 (53.1)	348 (54.5)
Female	7,118 (45.9)	3145 (46.0)	2,880 (46.7)	759 (42.9)	273 (42.7)
Missing	637 (4.1)	285 (4.2)	259 (4.2)	70 (4.0)	18 (2.8)
Era					
1995–1999	2,545 (16.4)	1109 (16.2)	1,014 (16.4)	312 (17.6)	109 (17.1)
2000–2004	3,042 (19.6)	1344 (19.7)	1,273 (20.6)	299 (16.9)	126 (19.7)
2005–2009	3,496 (22.5)	1588 (23.2)	1,355 (22.0)	412 (23.3)	140 (21.9)
2010–2016	6,440 (41.5)	2798 (40.9)	2,524 (40.9)	746 (42.2)	264 (41.3)

Data were presented as N (%) unless stated otherwise; IQR 75^th^– 25^th^ percentile.

ATSI Aboriginal and Torres Strait Islanders.

### Recipient blood group O not associated with differences post-transplant patient survival

**Fig 1:**
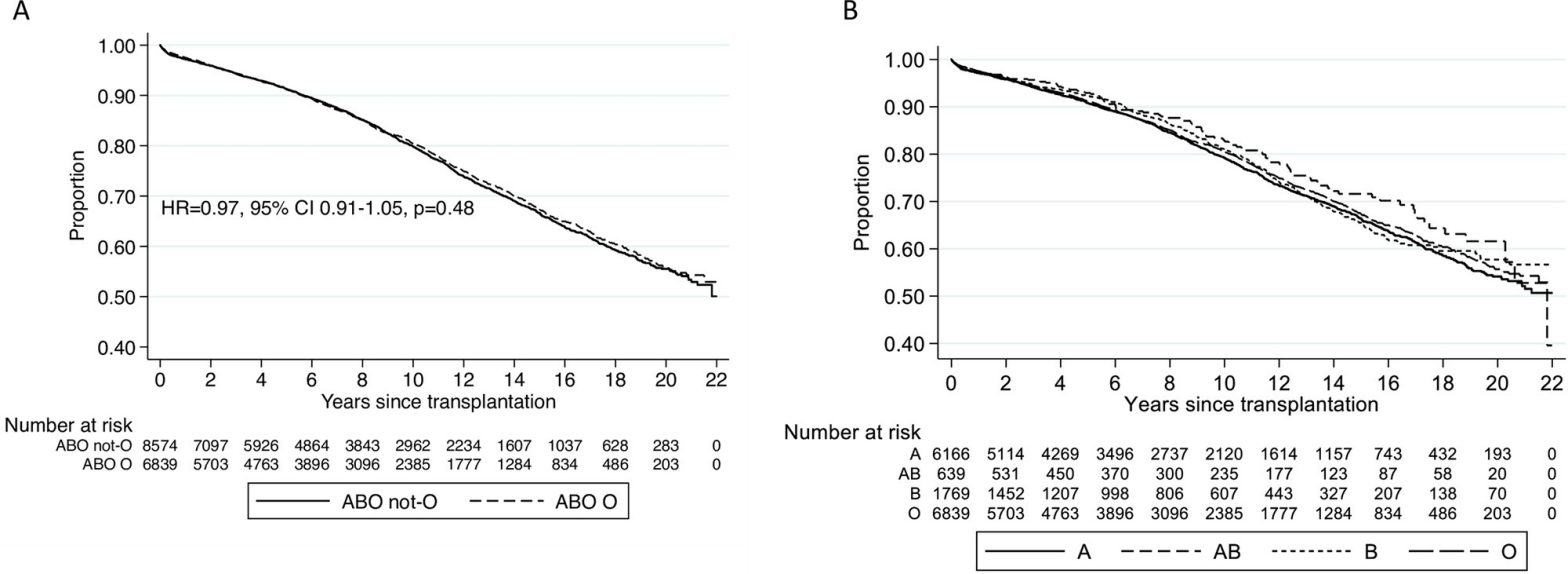
Kaplan Meier survival curve for cumulative patient survival post-transplant by recipient ABO blood group. (A) Recipient blood group O vs. non-blood group O. (B) All recipient blood groups.

Blood group O recipients had similar post-transplant survival compared to non-blood group O recipients (HR 0.96, 95% CI 0.89–1.04, Fig 1A, S2 Table). Increasing age at transplant, BMI <18.5, Aboriginal and Torres Strait Islander or Maori descent, any dialysis, any prior smoking, vascular disease, diabetes, respiratory disease and greater than 18 hour ischaemia time were linked to reduced patient survival than their counterparts (S2 Table). Asian descent, glomerulonephritis, polycystic kidney disease and reflux nephropathy were linked to increased patient survival. Blood group A, B and AB recipients had similar post-transplant survivals compared to blood group O recipients (Fig 1B, S3 Table).

**Fig 2:**
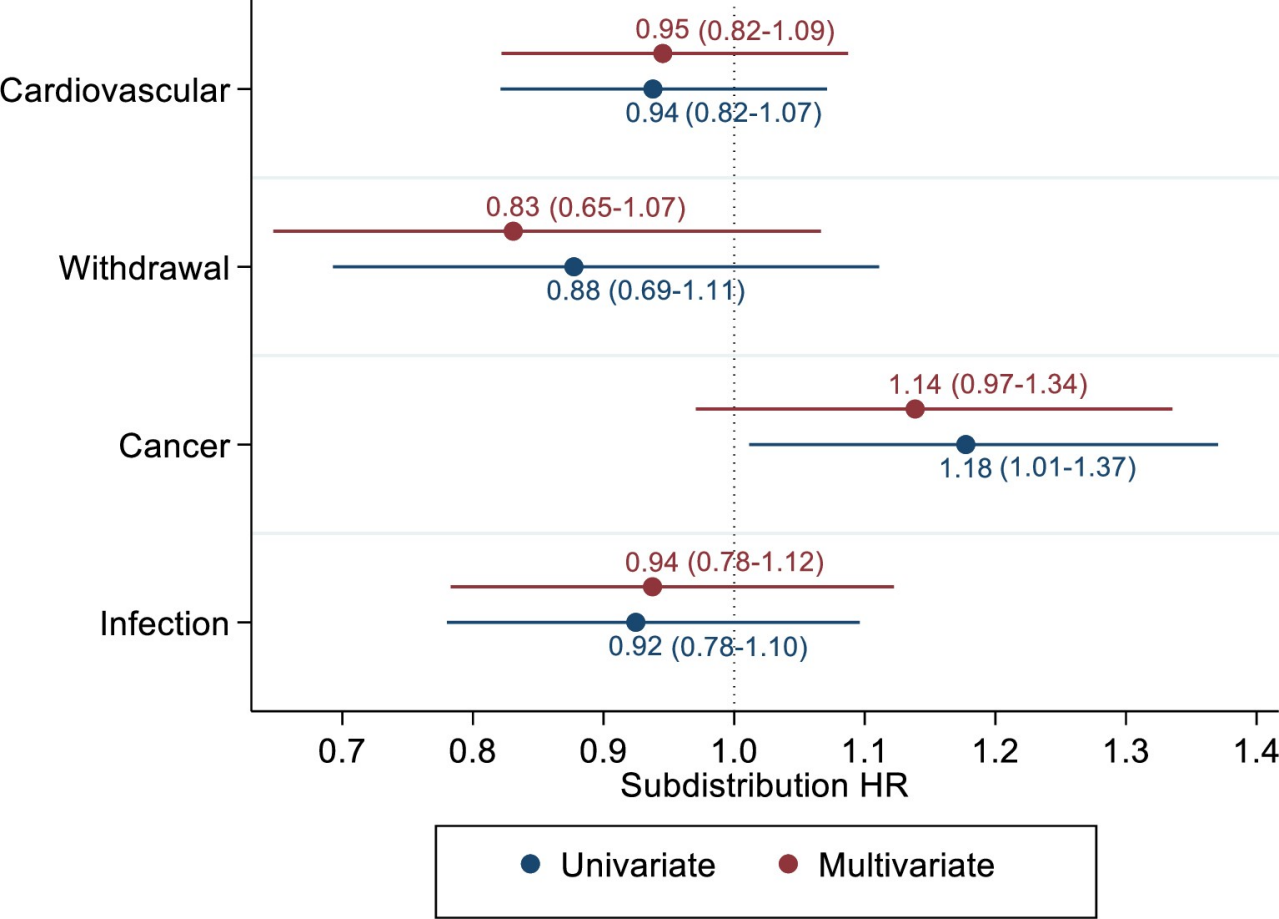
Univariate and multivariate competing risk analysis of cause-specific patient mortality between O and non-O ABO group. The estimates show the subdistribution hazards ratio for ABO O group where non-O ABO group is the reference point.

On competing risk analyses, blood group O was associated with 18% increased risk of cancer-related mortality (HR 1.18, 95% CI 1.01–1.37, Fig 2). However, this became statistically insignificant on multivariate analyses (HR 1.14, 95% CI 0.97–1.34). ABO blood group were not associated with differences in mortality from cardiovascular disease, infection nor withdrawal from dialysis.

### Recipient blood group AB associated with inferior death censored graft survival

**Fig 3:**
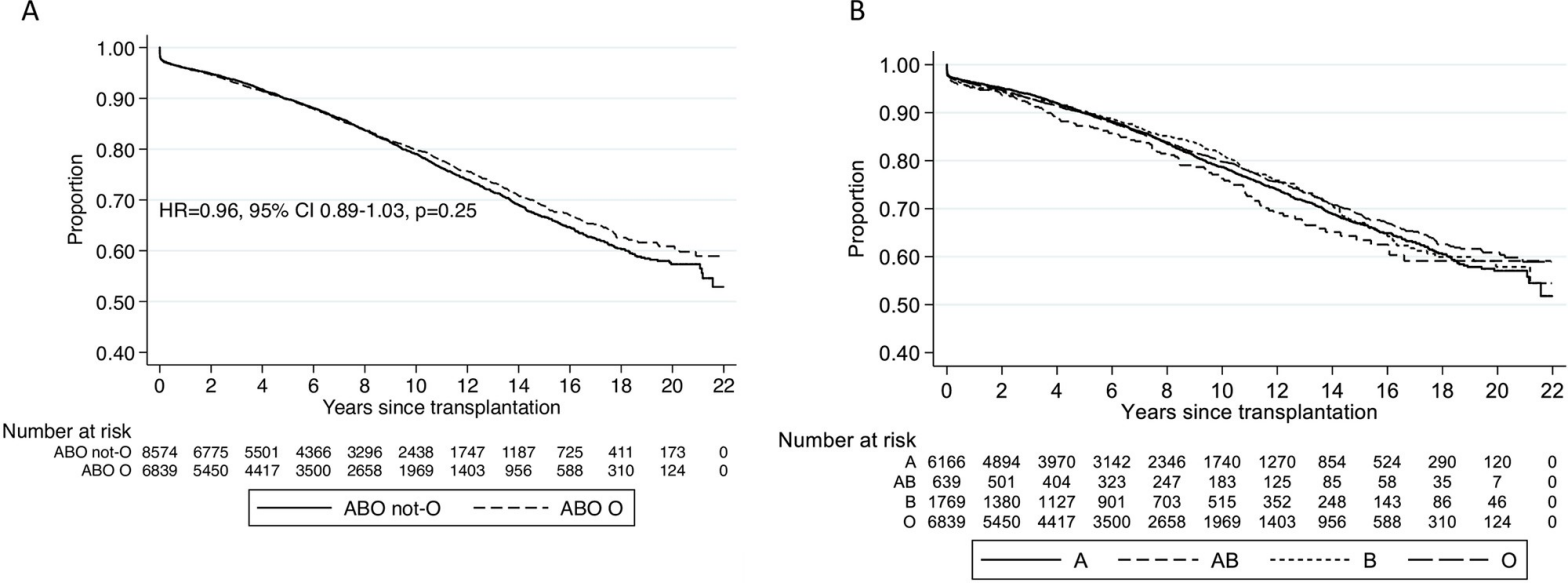
Kaplan Meier survival curve for death censored graft survival by recipient ABO blood group. (A) Recipient blood group O vs. non-blood group O. (B) All recipient blood groups.

While there was no difference in death censored graft survival between blood group O and non-O blood groups (HR 0.97, 95% CI 0.89–1.05, Fig 3A, S4 Table), blood group AB recipient had inferior death censored graft survival compared to blood group O (HR 1.24, 95% CI 1.02–1.50; S5 Table, Fig 3B). BMI ≥30, Aboriginal and Torres Strait Islander, Maori or Pacific descent, dialysis greater than 2 years, current smoking, vascular disease, diabetes, ≥3 HLA mismatches, increasing donor age >50 years old were linked to reduced graft survival (S5 Table). Later transplant vintage was associated with improved graft survival.

## Discussion

On analysis of 15, 523 kidney transplant patients in Australia and New Zealand between 1995–2016, recipient blood group O had similar patient survival post-transplant compared to non-O blood groups. This is similar to findings in 854 lung transplant patients where recipient ABO blood group was not an independent risk factor for one-year post-transplant survival [18]. These results suggest that ABO blood group antigen may not significantly affect post-transplant survival. Conversely in 557 liver transplant patients, recipient blood group O was associated with reduced post-transplant survival compared to non-O blood groups (HR 1.75, 95% CI 1.04–2.87, p = 0.035) [19]. However, patients with blood group O are known to have longer waitlist times for liver transplant compared to those with non-O blood groups—potentially contributing to the observed reduced patient survival [20].

Competing risks analyses demonstrated that blood group O were at increased risk of cancer-related mortality compared to non-blood group O recipients on univariate analyses. This may be associated with increased risks of skin cancer and non-hodgkin’s lymphoma in blood group O patients [10–12]—both of which are linked to higher risks of cancer-related mortality in Australian and New Zealand renal transplant patients between 1980 to 2014 [21]. Additionally, blood group O recipients had the longest pre-transplant dialysis duration. Dialysis has been associated with increased risks of oral, colorectal, liver, blood, breast, renal, upper urinary tract, and bladder cancer in population cohort studies [22, 23]. This could be due to dialysis-related immune dysfunction however, no differences in infection related mortality were observed. It is more likely that the combination of chronic infection, inflammation, immune dysfunction, malnutrition, and shortage of DNA repair mechanisms associated with dialysis promotes tumorigenesis [24, 25]. Unfortunately, data regarding the type of cancer was not available and is a limitation of this study. Further studies are required to investigate the effect of recipient ABO blood groups on the incidence of cancer subtypes post-transplant.

Recipient blood group AB was associated reduced death censored graft survival compared to blood group O. Notably, recipients with AB blood group only comprised 4.11% (639/15,523) of kidney transplants; and had different ethnicity distribution, pre-transplant dialysis duration, prolonged total ischemia time, donor type, donor age and transplant era compared to non-AB blood group recipients. Furthermore, it is a highly heterogenous population in terms of duration of pre-transplant renal replacement therapy, recipient and donor factors owing to the variable supply of donor AB blood group kidney grafts [26]. As a result, the inferior death-censored graft survival observed in this study may not be generalized to all AB blood groups recipients.

Moreover, recipient blood group may be a surrogate marker for established adverse outcome factors. This is supported by differences in recipient BMI, ethnicity, pre-transplant renal replacement therapy, total ischaemia time, HLA mismatch and donor type by recipient blood group. For example, pre-transplant obesity is associated with increased mortality and death-censored graft survival in a meta-analysis of 10 adult studies [27]. Aboriginal and Torres Strait Islander recipients have prolonged wait times, increased number of HLA mismatches, enhanced socioeconomic barriers to ongoing treatment and shortened recipient survival compared to non-Aboriginal and Torres Strait Islander Australians [28]. However, differences in survival in Aboriginal and Torres Strait Islander Australians have primarily been attributed to infective causes which was not observed in this study [29]. Pre-emptive kidney transplants and living donor grafts are associated with improved patient and graft survival [30].

Increasing recipient age, longer duration of pre-transplant renal replacement therapy, current smoking status, vascular disease, diabetes mellitus, deceased donor and early era of transplant are independent risk factors for adverse post-transplant patient outcomes per meta-analyses [31] along with, database analyses of US [32–35], Australian, New Zealand [36], and Dutch [30] recipients.

The major strength of this study is the use of a large registry with almost complete capture from time of dialysis initiation to graft loss and/or mortality. However, the use of observational data carries inherent risks related to confounding factors. Multivariate analyses can be used to control for known confounders however, it cannot address unmeasured confounders such as socioeconomic status, nutrition and environmental exposures. It follows that blood group A may be a surrogate marker for an unmeasured factor such as geographic location, family history of heart disease or financial status.

Additionally, the findings of this study are primarily applicable to the Australian and New Zealand population and health infrastructure. Further studies using other databases such as United States Renal Data System and United Kingdom Renal Registry could be completed to determine if the correlations observed in this study are translatable to other jurisdictions. Lastly, this study did not investigate if there was a link between rates of acute or chronic rejection and recipient ABO blood group. The effect of recipient ABO blood group on causes of death-censored graft failure were not investigated.

## Conclusions

Recipient blood group O was not associated with differences in all-cause mortality. There may be ABO blood group effects on cause-specific (e.g. cancer-related) mortality. Recipient blood group AB was associated with reduced death censored graft survival however, this is difficult to interpret in the context of the small highly heterogenous patient population included in this study. Further investigations in larger populations are required to clarify the effects of specific ABO blood groups on all-cause mortality and cause-specific mortality.

## Supporting information

S1 Table ABO-compatible (●) and ABO-incompatible transplants (○). (DOCX)

S2 Table Univariate and multivariate Cox proportional hazard model of patients’ survivals between O and non-O ABO group. (DOCX)

S3 Table Univariate and multivariate Cox proportional hazard model of patients’ survivals between A, B, AB and O groups. (DOCX)

S4 Table Univariate and multivariate Cox proportional hazard model of death censored graft survivals between O and non-O ABO group. (DOCX)

S5 Table Univariate and multivariate Cox proportional hazard model of death censored graft survivals between A, B, AB and O groups. (DOCX)
