# Supplementary material for: ABO blood group relationships to kidney transplant recipient and graft outcomes
Source: PLoS One. 2020 Jul 23;15(7):e0236396. doi: 10.1371/journal.pone.0236396 (PMC7377395; doi:10.1371/journal.pone.0236396)
Supplement: S1 Table — (DOCX) [file pone.0236396.s001.docx]

**S1 Table:** ABO-compatible (●) and ABO-incompatible transplants (○)

|  |  | **Recipient** | | | |
| --- | --- | --- | --- | --- | --- |
|  |  | **O** | **A** | **B** | **AB** |
| **Donor** | **O** | ● | ● | ● | ● |
|  | **A** | ○ | ● | ○ | ● |
|  | **B** | ○ | ○ | ● | ● |
|  | **AB** | ○ | ○ | ○ | ● |
