# Supplementary material for: ABO blood group relationships to kidney transplant recipient and graft outcomes
Source: PLoS One. 2020 Jul 23;15(7):e0236396. doi: 10.1371/journal.pone.0236396 (PMC7377395; doi:10.1371/journal.pone.0236396)
Supplement: S2 Table — (DOCX) [file pone.0236396.s002.docx]

S2 Table: Univariate and multivariate Cox proportional hazard model of patients’ survivals between O and non-O ABO group

| Characteristics | | | | | Univariate model | | Multivariate model | | | |
| --- | --- | --- | --- | --- | --- | --- | --- | --- | --- | --- |
|  | | | | | HR (95% CI) | P Value | HR (95% CI) | P Value | | |
| ABO Blood group | | | | |  |  |  |  | | |
|  | | Group non-O  Group O | | | Reference  0.97 (0.91-1.05) | -  0.48 | Reference  0.96 (0.89-1.04) | 0.35 | | |
| Age at transplant, y* | | | | |  |  |  |  | | |
|  | | <=40y (per year) | | | 1.09 (1.08-1.10) | <0.001 | 1.03 (1.02-1.04) | <0.001 | | |
|  | | >40y (per year) | | | 1.07 (1.07-1.08) | <0.001 | 1.06 (1.05-1.06) | <0.001 | | |
| Gender | | | | | |  |  |  | | |
|  | Male | | | | Reference | - | Reference |  | | |
|  | Female | | | | 0.88 (0.82-0.95) | <0.01 | 0.97 (0.89-1.05) | 0.41 | | |
| BMI, kg/m2 | | | | | |  |  |  | | |
|  | | <18.5 | | | 0.90 (0.72-1.13) | 0.37 | 1.29 (1.01-1.64) | 0.03 | | |
|  |  | 18.5-24.9 | | | Reference | - | Reference |  | | |
|  |  | 25-29.9 | | | 1.25 (1.15-1.36) | <0.001 | 0.92 (0.84-1.00) | 0.047 | | |
|  |  | >=30 | | | 1.46 (1.32-1.61) | <0.001 | 1.00 (0.90-1.11) | 0.96 | | |
| Ethnicity | | | | |  |  |  |  | | |
|  | | White | | | Reference |  |  |  | | |
|  | | Aboriginal/TSI | | | 2.56 (2.20-2.98) | <0.001 | 1.74 (1.45-2.10) | <0.001 | | |
|  | | Asian | | | 0.82 (0.71-0.94) | <0.01 | 0.76 (0.65-0.88) | <0.001 | | |
|  | | Maori | | | 1.84 (1.53-2.21) | <0.001 | 1.26 (1.01-1.57) | 0.04 | | |
|  | | Pacific | | | 0.92 (0.72-1.19) | 0.54 | 0.96 (0.74-1.25) | 0.78 | | |
|  | | Other/Not reported | | | 0.49 (0.32-0.77) | <0.01 | 0.64 (0.42-0.98) | 0.04 | | |
| Primary renal disease | | | | |  |  |  |  | | |
|  | | Diabetic Nephropathy | | | Reference | - | Reference |  | | |
|  | | Glomerulonephritis | | | 0.42 (0.38-0.46) | <0.001 | 0.62 (0.51-0.75) | <0.001 | | |
|  | | Hypertension | | | 0.89 (0.76-1.05) | 0.17 | 0.80 (0.64-1.01) | 0.06 | | |
|  | | Polycystic Disease | | | 0.53 (0.47-0.61) | <0.001 | 0.63 (0.51-0.78) | <0.001 | | |
|  | | Reflux Nephropathy | | | 0.29 (0.24-0.34) | <0.001 | 0.62 (0.48-0.79) | <0.001 | | |
|  | | Other/Not reported | | | 0.52 (0.46-0.59) | <0.001 | 0.86 (0.70-1.06) | 0.16 | | |
| Dialysis duration, y | | | | |  |  |  |  | | |
|  | | | Pre-emptive | | Reference |  |  |  | | |
|  | | | ≤ 1 | | 1.84 (1.55-2.18) | <0.001 | 1.25 (1.05-1.49) | 0.01 | | |
|  | | | 2-3 | | 3.26 (2.75-3.88) | <0.001 | 1.74 (1.45-2.08) | <0.001 | | |
|  | | | ≥ 4 | | 3.48 (2.92-4.14) | <0.001 | 1.81 (1.50-2.19) | <0.001 | | |
| Smoking status | | | | |  |  |  |  | | |
|  | Never | | | | Reference | - | Reference | - | | |
|  | Former | | | | 1.63 (1.50-1.76) | <0.001 | 1.14 (1.05-1.24) | <0.01 | | |
|  | Current | | | | 1.88 (1.70-2.09) | <0.001 | 1.63 (1.45-1.83) | <0.001 | | |
| Vascular disease | | | |  | |  |  | | |  |
|  | | No | | Reference | | - | Reference | | | - |
|  | | Yes | | 2.84 (2.63-3.06) | | <0.001 | 1.45 (1.33-1.59) | | | <0.001 |
| Diabetes | | | |  | |  |  | |  | |
|  | | No | | Reference | | - | Reference | | - | |
|  | | Yes | | 2.33 (2.15-2.53) | | <0.001 | 1.34 (1.13-1.59) | | <0.01 | |
| Respiratory disease | | | |  | |  |  | |  | |
|  | | No  Yes | | Reference  2.09 (1.85-2.37) | | <0.001 | Reference  1.25 (1.08-1.46) | | -  <0.01 | |
| Total ischemia time (hour) | | | |  | |  |  | |  | |
|  | | <12h | | Reference | | - | Reference | |  | |
|  | | 12h-18h | | 1.64 (1.52-1.78) | | <0.001 | 1.02 (0.92-1.13) | | 0.73 | |
|  | | 18h+ | | 2.07 (1.86-2.30) | | <0.001 | 1.17 (1.03-1.33) | | 0.02 | |
| HLA Mismatches | | | |  | |  |  | |  | |
|  | | 0 | | Reference | |  | Reference | |  | |
|  | | 1 | | 1.31 (1.07-1.59) | | <0.01 | 0.99 (0.81-1.21) | | 0.94 | |
|  | | 2 | | 1.25 (1.05-1.50) | | 0.01 | 1.11 (0.93-1.33) | | 0.26 | |
|  | | 3 | | 1.19 (0.99-1.42) | | 0.06 | 1.07 (0.89-1.28) | | 0.46 | |
|  | | 4 | | 1.62 (1.35-1.95) | | <0.001 | 1.21 (1.00-1.45) | | 0.045 | |
|  | | 5 | | 1.67 (1.40-2.00) | | <0.001 | 1.17 (0.98-1.41) | | 0.09 | |
|  | | 6 | | 1.68 (1.38-2.05) | | <0.001 | 1.19 (0.97-1.47) | | 0.10 | |
| Type of Donors | | | |  | |  |  | |  | |
|  | | Live | | Reference | | - | Reference | | - | |
|  | | Deceased | | 2.00 (1.84-2.18) | | <0.001 | 1.07 (0.95-1.20) | | 0.24 | |
| Donor age, y | | | |  | |  |  | |  | |
|  | | <=50y (per year) | | 1.02 (1.02-1.02) | | <0.001 | 1.02 (1.01-1.02) | | <0.001 | |
|  | | >50y (per year) | | 1.04 (1.04-1.05) | | <0.001 | 1.01 (1.00-1.02) | | <0.01 | |
| Donor gender | | | |  | |  |  | |  | |
|  | | Male | | Reference | | - | - | | - | |
|  | | Female | | 0.99 (0.92-1.06) | | 0.81 | - | | - | |
| Era | | | |  | |  |  | |  | |
|  | | 1995-1999 | | Reference | | - | Reference | | - | |
|  | | 2000-2004 | | 0.82 (0.75-0.90) | | <0.001 | 0.71 (0.64-0.78) | | <0.001 | |
|  | | 2005-2009 | | 0.77 (0.69-0.86) | | <0.001 | 0.56 (0.50-0.63) | | <0.001 | |
|  | | 2010-2016 | | 0.73 (0.64-0.84) | | <0.001 | 0.40 (0.34-0.47) | | <0.001 | |

TSI, Torres Strait Islander; BMI, body mass index; CI, confidence Interval; HR, Hazard ratio; HLA, human leukocyte antigen; y, years old
