# Supplementary material for: ABO blood group relationships to kidney transplant recipient and graft outcomes
Source: PLoS One. 2020 Jul 23;15(7):e0236396. doi: 10.1371/journal.pone.0236396 (PMC7377395; doi:10.1371/journal.pone.0236396)
Supplement: S3 Table — (DOCX) [file pone.0236396.s003.docx]

S3 Table: Univariate and multivariate Cox proportional hazard model of patients’ survivals between A, B, AB and O groups

| Characteristics | | | | Univariate model | | Multivariate model | |
| --- | --- | --- | --- | --- | --- | --- | --- |
|  | | | | HR (95% CI) | P Value | HR (95% CI) | P Value |
| ABO Blood group | | | |  |  |  |  |
|  | | A  AB | | 1.05 (0.98-1.14)  0.88 (0.72-1.06) | 0.18  0.18 | 1.07 (0.99 – 1.16)  0.90 (0.74-1.10) | 0.11  0.31 |
|  | | B | | 0.98 (0.87-1.11) | 0.79 | 0.96 (0.85-1.09) | 0.56 |
|  | | O | | Reference | - | Reference | - |
| Age at transplant, y* | | | |  |  |  |  |
|  | | <=40y (per year) | | 1.09 (1.08-1.10) | <0.001 | 1.03 (1.02-1.04) | <0.001 |
|  | | >40y (per year) | | 1.07 (1.07-1.08) | <0.001 | 1.06 (1.05-1.06) | <0.001 |
| Gender | | | | |  |  |  |
|  | Male | | | Reference | - | Reference |  |
|  | Female | | | 0.88 (0.82-0.95) | <0.01 | 0.97 (0.89-1.05) | 0.41 |
| BMI, kg/m2 | | | | |  |  |  |
|  | | <18.5 | | 0.90 (0.72-1.13) | 0.37 | 1.30 (1.02-1.65) | 0.04 |
|  |  | 18.5-24.9 | | Reference | - | Reference |  |
|  |  | 25-29.9 | | 1.25 (1.15-1.36) | <0.001 | 0.92 (0.84-1.00) | 0.05 |
|  |  | >=30 | | 1.46 (1.32-1.61) | <0.001 | 1.00 (0.90-1.11) | 0.96 |
| Ethnicity | | | |  |  |  |  |
|  | | White | | Reference |  |  |  |
|  | | Aboriginal/TSI | | 2.56 (2.20-2.98) | <0.001 | 1.73 (1.44-2.08) | <0.001 |
|  | | Asian | | 0.82 (0.71-0.94) | <0.01 | 0.78 (0.67-0.91) | <0.01 |
|  | | Maori | | 1.84 (1.53-2.21) | <0.001 | 1.25 (1.00-1.56) | 0.049 |
|  | | Pacific | | 0.92 (0.72-1.19) | 0.54 | 0.97 (0.75-1.26) | 0.84 |
|  | | Other/Not reported | | 0.49 (0.32-0.77) | <0.01 | 0.65 (0.43-0.99) | 0.047 |
| Primary renal disease | | | |  |  |  |  |
|  | | Diabetic Nephropathy | | Reference | - | Reference |  |
|  | | Glomerulonephritis | | 0.42 (0.38-0.46) | <0.001 | 0.62 (0.51-0.75) | <0.001 |
|  | | Hypertension | | 0.89 (0.76-1.05) | 0.17 | 0.80 (0.64-1.01) | 0.06 |
|  | | Polycystic Disease | | 0.53 (0.47-0.61) | <0.001 | 0.63 (0.51-0.78) | <0.001 |
|  | | Reflux Nephropathy | | 0.29 (0.24-0.34) | <0.001 | 0.62 (0.48-0.79) | <0.001 |
|  | | Other/Not reported | | 0.52 (0.46-0.59) | <0.001 | 0.86 (0.70-1.06) | 0.15 |
| Dialysis duration, y | | | |  |  |  |  |
|  | | | Pre-emptive | Reference |  |  |  |
|  | | | ≤ 1 | 1.84 (1.55-2.18) | <0.001 | 1.25 (1.05-1.49) | 0.01 |
|  | | | 2-3 | 3.26 (2.75-3.88) | <0.001 | 1.73 (1.44-2.07) | <0.001 |
|  | | | ≥ 4 | 3.48 (2.92-4.14) | <0.001 | 1.80 (1.49-2.17) | <0.001 |
| Smoking status | | | |  |  |  |  |
|  | Never | | | Reference | - | Reference | - |
|  | Former | | | 1.63 (1.50-1.76) | <0.001 | 1.15 (1.05-1.25) | <0.01 |
|  | Current | | | 1.88 (1.70-2.09) | <0.001 | 1.64 (1.46-1.84) | <0.001 |

**S3 Table**. (Continued….)

| Characteristics | | | Univariate model | | Multivariate model | |
| --- | --- | --- | --- | --- | --- | --- |
|  | | | HR (95% CI) | P Value | HR (95% CI) | P Value |
| Vascular disease | | |  |  |  |  |
|  | | No | Reference | - | Reference | - |
|  | | Yes | 2.84 (2.63-3.06) | <0.001 | 1.45 (1.33-1.59) | <0.001 |
| Diabetes | | |  |  |  |  |
|  | No | | Reference | - | Reference | - |
|  | Yes | | 2.33 (2.15-2.53) | <0.001 | 1.34 (1.13-1.59) | <0.01 |
| Respiratory disease | | |  |  |  |  |
|  | No  Yes | | Reference  2.09 (1.85-2.37) | <0.001 | Reference  1.26 (1.08-1.46) | -  <0.01 |
| Total ischemia time (hour) | | |  |  |  |  |
|  | <12h | | Reference | - | Reference |  |
|  | 12h-18h | | 1.64 (1.52-1.78) | <0.001 | 1.02 (0.92-1.13) | 0.71 |
|  | 18h+ | | 2.07 (1.86-2.30) | <0.001 | 1.17 (1.03-1.33) | 0.02 |
| HLA Mismatches | | |  |  |  |  |
|  | 0 | | Reference |  | Reference |  |
|  | 1 | | 1.31 (1.07-1.59) | <0.01 | 0.99 (0.81-1.21) | 0.95 |
|  | 2 | | 1.25 (1.05-1.50) | 0.01 | 1.11 (0.93-1.33) | 0.24 |
|  | 3 | | 1.19 (0.99-1.42) | 0.06 | 1.07 (0.90-1.29) | 0.44 |
|  | 4 | | 1.62 (1.35-1.95) | <0.001 | 1.22 (1.02-1.47) | 0.03 |
|  | 5 | | 1.67 (1.40-2.00) | <0.001 | 1.19 (0.99-1.43) | 0.07 |
|  | 6 | | 1.68 (1.38-2.05) | <0.001 | 1.20 (0.97-1.48) | 0.09 |
| Type of Donors | | |  |  |  |  |
|  | Live | | Reference | - | Reference | - |
|  | Deceased | | 2.00 (1.84-2.18) | <0.001 | 1.08 (0.96-1.21) | 0.20 |
| Donor age, y | | |  |  |  |  |
|  | <=50y (per year) | | 1.02 (1.02-1.02) | <0.001 | 1.02 (1.01-1.02) | <0.001 |
|  | >50y (per year) | | 1.04 (1.04-1.05) | <0.001 | 1.01 (1.00-1.02) | 0.01 |
| Era | | |  |  |  |  |
|  | 1995-1999 | | Reference | - | Reference | - |
|  | 2000-2004 | | 0.82 (0.75-0.90) | <0.001 | 0.71 (0.64-0.78) | <0.001 |
|  | 2005-2009 | | 0.77 (0.69-0.86) | <0.001 | 0.56 (0.50-0.63) | <0.001 |
|  | 2010-2016 | | 0.73 (0.64-0.84) | <0.001 | 0.40 (0.34-0.46) | <0.001 |

TSI, Torres Strait Islander; BMI, body mass index; CI, confidence Interval; HR, Hazard ratio; HLA, human leukocyte antigen; y, years old
