# Supplementary material for: ABO blood group relationships to kidney transplant recipient and graft outcomes
Source: PLoS One. 2020 Jul 23;15(7):e0236396. doi: 10.1371/journal.pone.0236396 (PMC7377395; doi:10.1371/journal.pone.0236396)
Supplement: S4 Table — (DOCX) [file pone.0236396.s004.docx]

S4 Table: Univariate and multivariate Cox proportional hazard model of death censored graft survivals between O and non-O ABO group

| Characteristics | | | | | Univariate model | | Multivariate model | | | |
| --- | --- | --- | --- | --- | --- | --- | --- | --- | --- | --- |
|  | | | | | HR (95% CI) | P Value | HR (95% CI) | P Value | | |
| ABO Blood group | | | | |  |  |  |  | | |
|  | | Group non-O  Group O | | | Reference  0.96 (0.89-1.03) | -  0.25 | Reference  0.97 (0.89-1.05) | 0.41 | | |
| Age at transplant, y* | | | | |  |  |  |  | | |
|  | | <=40y (per year) | | | 0.98 (0.97-0.98) | <0.001 | 0.97 (0.96-0.98) | <0.001 | | |
|  | | >40y (per year) | | | 0.98 (0.98-0.99) | <0.001 | 0.99 (0.98-0.99) | <0.001 | | |
| Gender | | | | | |  |  |  | | |
|  | Male | | | | Reference | - | - | - | | |
|  | Female | | | | 1.01 (0.94-1.10) | 0.72 | - | - | | |
| BMI, kg/m2 | | | | | |  |  |  | | |
|  | | <18.5 | | | 1.28 (1.05-1.56) | 0.01 | 1.07 (0.87-1.33) | 0.51 | | |
|  |  | 18.5-24.9 | | | Reference | - | Reference |  | | |
|  |  | 25-29.9 | | | 0.97 (0.89-1.06) | 0.52 | 1.04 (0.94-1.14) | 0.45 | | |
|  |  | >=30 | | | 1.14 (1.03-1.27) | 0.01 | 1.23 (1.10-1.37) | 0.96 | | |
| Ethnicity | | | | |  |  |  |  | | |
|  | | White | | | Reference |  |  |  | | |
|  | | Aboriginal/TSI | | | 2.65 (2.23-3.15) | <0.001 | 1.87 (1.55-2.25) | <0.001 | | |
|  | | Asian | | | 1.06 (0.93-1.22) | 0.39 | 0.99 (0.86-1.15) | 0.93 | | |
|  | | Maori | | | 1.96 (1.60-2.40) | <0.001 | 1.67 (1.36-2.06) | <0.001 | | |
|  | | Pacific | | | 2.08 (1.72-2.52) | <0.001 | 1.76 (1.43-2.17) | <0.001 | | |
|  | | Other/Not reported | | | 0.73 (0.50-1.06) | 0.10 | 0.69 (0.47-1.03) | 0.07 | | |
| Primary renal disease | | | | |  |  |  |  | | |
|  | | Diabetic Nephropathy | | | Reference | - | Reference |  | | |
|  | | Glomerulonephritis | | | 0.88 (0.78-0.99) | 0.04 | 1.18 (0.93-1.49) | 0.17 | | |
|  | | Hypertension | | | 0.77 (0.62-0.96) | 0.02 | 1.12 (0.82-1.51) | 0.49 | | |
|  | | Polycystic Disease | | | 0.48 (0.40-0.57) | <0.001 | 0.79 (0.60-1.04) | 0.09 | | |
|  | | Reflux Nephropathy | | | 0.96 (0.82-1.12) | 0.58 | 1.27 (0.98-1.64) | 0.07 | | |
|  | | Other/Not reported | | | 0.97 (0.85-1.11) | 0.68 | 1.12 (0.87-1.44) | 0.36 | | |
| Dialysis duration, y | | | | |  |  |  |  | | |
|  | | | Pre-emptive | | Reference |  |  |  | | |
|  | | | ≤ 1 | | 1.20 (1.04-1.37) | <0.01 | 1.10 (0.96-1.27) | 0.18 | | |
|  | | | 2-3 | | 1.37 (1.19-1.58) | <0.001 | 1.29 (1.10-1.50) | <0.01 | | |
|  | | | ≥ 4 | | 1.61 (1.40-1.86) | <0.001 | 1.48 (1.25-1.74) | <0.001 | | |
| Smoking status | | | | |  |  |  |  | | |
|  | Never | | | | Reference | - | Reference | - | | |
|  | Former | | | | 0.99 (0.91-1.08) | 0.88 | 1.10 (1.00-1.21) | 0.045 | | |
|  | Current | | | | 1.53 (1.37-1.71) | <0.001 | 1.46 (1.30-1.65) | <0.001 | | |
| Vascular disease | | | |  | |  |  | | |  |
|  | | No | | Reference | | - | Reference | | | - |
|  | | Yes | | 1.17 (1.06-1.29) | | <0.01 | 1.23 (1.09-1.38) | | | <0.01 |
| Diabetes | | | |  | |  |  | |  | |
|  | | No | | Reference | | - | Reference | | - | |
|  | | Yes | | 1.25 (1.13-1.39) | | <0.001 | 1.40 (1.13-1.73) | | <0.01 | |
| Respiratory disease | | | |  | |  |  | |  | |
|  | | No  Yes | | Reference  1.12 (0.95-1.33) | | 0.17 | Reference  1.06 (0.88-1.26) | | -  0.55 | |
| Total ischemia time (hour) | | | |  | |  |  | |  | |
|  | | <12h | | Reference | | - | Reference | |  | |
|  | | 12h-18h | | 1.12 (1.03-1.22) | | <0.01 | 0.95 (0.84-1.06) | | 0.34 | |
|  | | 18h+ | | 1.42 (1.26-1.60) | | <0.001 | 1.07 (0.92-1.24) | | 0.40 | |
| HLA Mismatches | | | |  | |  |  | |  | |
|  | | 0 | | Reference | |  | Reference | |  | |
|  | | 1 | | 1.45 (1.16-1.82) | | <0.01 | 1.17 (0.93-1.48) | | 0.19 | |
|  | | 2 | | 1.53 (1.25-1.87) | | <0.001 | 1.25 (1.01-1.55) | | 0.04 | |
|  | | 3 | | 1.70 (1.39-2.07) | | <0.001 | 1.34 (1.09-1.65) | | <0.01 | |
|  | | 4 | | 1.82 (1.48-2.23) | | <0.001 | 1.54 (1.24-1.92) | | <0.001 | |
|  | | 5 | | 1.83 (1.50-2.25) | | <0.001 | 1.59 (1.28-1.97) | | <0.001 | |
|  | | 6 | | 2.00 (1.61-2.50) | | <0.001 | 1.69 (1.34-2.14) | | <0.001 | |
| Type of Donors | | | |  | |  |  | |  | |
|  | | Live | | Reference | | - | Reference | | - | |
|  | | Deceased | | 1.17 (1.08-1.27) | | <0.001 | 0.85 (0.75-0.96) | | 0.01 | |
| Donor age, y | | | |  | |  |  | |  | |
|  | | <=50y (per year) | | 1.03 (1.03-1.03) | | <0.001 | 1.02 (1.01-1.02) | | <0.001 | |
|  | | >50y (per year) | | 1.04 (1.04-1.05) | | <0.001 | 1.03 (1.03-1.04) | | <0.001 | |
| Donor gender | | | |  | |  |  | |  | |
|  | | Male | | Reference | | - | - | | - | |
|  | | Female | | 1.08 (1.00-1.17) | | 0.04 | 1.01 (0.94-1.10) | | 0.72 | |
| Era | | | |  | |  |  | |  | |
|  | | 1995-1999 | | Reference | | - | Reference | | - | |
|  | | 2000-2004 | | 0.80 (0.72-0.88) | | <0.001 | 0.72 (0.65-0.80) | | <0.001 | |
|  | | 2005-2009 | | 0.73 (0.66-0.82) | | <0.001 | 0.54 (0.48-0.61) | | <0.001 | |
|  | | 2010-2016 | | 0.59 (0.51-0.67) | | <0.001 | 0.42 (0.36-0.49) | | <0.001 | |

TSI, Torres Strait Islander; BMI, body mass index; CI, confidence Interval; HR, Hazard ratio; HLA, human leukocyte antigen; y, years old
